# Primary Infection with Early SARS-CoV-2 (D614G) Induces Cross Neutralization Antibodies against Omicron BA.5 and EG.5.1

**DOI:** 10.24546/0100495531

**Published:** 2025-04-02

**Authors:** MARIA ISTIQOMAH MARINI, REI TAKAMIYA, MITSUHIRO NISHIMURA, YASUKO MORI

**Affiliations:** 1 Division of Clinical Virology, Center for Infectious Diseases, Kobe University Graduate School of Medicine, Kobe, Japan

**Keywords:** COVID-19, Critical patients, Neutralizing antibody, SARS-CoV-2, Omicron variants

## Abstract

SARS-CoV-2 Omicron sub-variants are still emerging and become highly diversified, resulting in increased transmissibility and immune evasion ability. Neutralizing antibody is very important to fight against the variants. However, the ability of neutralizing antibody induced by early SARS-CoV-2 (D614G) primary infection has not been analyzed in detail against lately emerged Omicron variants, such as BA.5 and EG.5.1. In this study the sera from severe/critically infected patients of D614G were investigated the potency of neutralization activity against SARS-CoV-2 BA.5 and EG.5.1 by using live virus neutralization assay. The neutralizing activity was observed and analyzed in detail from day 1 until 7 post infection. The sera of both severe and critical patients showed cross neutralizing activity for BA.5, and even for EG.5.1. It is suggested that neutralizing antibodies targeting conserved epitopes are partly induced upon the primary infection as the result of robust immune response.

## INTRODUCTION

Coronavirus disease 2019 (COVID-19) is a respiratory disease induced by the β-coronavirus, severe acute respiratory syndrome coronavirus 2 (SARS-CoV-2), which is responsible for the pandemic declared by the World Health Organization (WHO) on March, 2020 (1). Although the WHO emergency committee suggested that the COVID-19 situation is no longer global emergency on May 2023 (2), SARS-CoV-2 has been adapted into human society as other human coronaviruses. COVID-19 has symptoms that vary from complete asymptomatic cases to symptomatic with mild, moderate, severe, and critical illness (3). Infected people manifest upper respiratory symptoms in one to five days post infection and then sometimes develop pneumonia. The pneumonia patients are classified into three groups according to the severity: moderate: oxygen saturation levels over 90% on room air, severe: oxygen saturation level under 90% on room air, and critical: who need mechanical ventilation (4, 5). Direct injury to organs by the virus infection as well as the uncontrollable immune response, known as the cytokine storm, lead to the life-threatening, extensive systemic damages on the patients (6).

Humoral immunity is essential for combating and safeguarding against SARS-CoV-2 infection and propagation (3). Neutralizing antibodies induced as the result of immune response to SARS-CoV-2 infection inhibit the infection, and thereby facilitate the clearance of the virus from the body (7). It has been revealed that the generation of neutralizing antibodies occurred prior to the undetectability of the SARS-CoV-2 genome, indicating effective viral clearance by successful antibody induction. The concentrations of several cytokines, chemokines, and growth factors remained elevated at the time when the viral genome PCR yielded a negative result. This finding indicates that the cytokine storm persists long after the virus has been eradicated, influencing the continued immune response and severity of COVID-19 (4). The high neutralizing antibody titer and various cytokines induced in critical patients may characterize unique immune response against SARS-CoV-2.

Since the pandemic of SARS-CoV-2, numerous variants have arisen, resulting in increased transmissibility and immune evasion ability (8). Firstly, SARS-CoV-2 acquired D614G mutation in the spike, and four variants of concern (VOCs) of SARS-CoV-2 were reported by the end of 2021, namely Alpha (B.1.1.7), Beta (B.1.351), Gamma (P.1), and Delta (B.1.617.2). These VOCs have additional mutations in the spike, thereby they escape from neutralizing antibodies targeting the surface antigen. The COVID-19 situation has drastically changed at the end of 2021 because of the emergence of the Omicron variants BA.1 and BA.2 which possess more than 30 mutations in the spike (9). The descendants of BA.2 has disseminated fast and BA.5 has spread across the globe, causing two large surges of COVID-19 in Japan around June 2022 and March 2023. Subsequently the sub-lineages of Omicron XBB variant dominated the world, and the one of descendant of the XBB, that is, EG.5.1 had spread in Japan around 2023 August.

**Figure 1:**
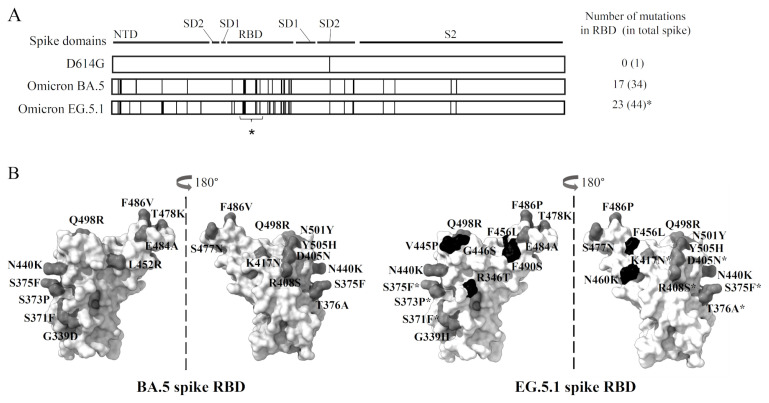
SARS-CoV-2 variants and mutations in the spike antigen. **A**. Amino acid mutation sites of SARS-CoV-2 variants D614G, Omicron BA.5, and EG.5.1 are indicated as bars. Structural domains of the spike are also shown above. The abbreviations are as follows; NTD: N-terminal domain, SD1: subdomain 1, SD2: subdomain 2, RBD: receptor binding domain. The mutations of EG.5.1 are from a reference sequence (EPI_ISL_17540065), although the live virus of EG.5.1 used in this study does not contain eight mutations at the site indicated by the asterisk. **B**. The surface presentations of RBD (amino acids 334–528) indicating mutation sites in the Omicron BA.5 and EG.5.1 variants. The RBD structure of Omicron BQ.1.1 spike (PDB ID: 8XI6) is used as reference (13). Mutation sites are indicated as grey and black colors. Black indicated mutation sites found in EG.5.1, but not in BA.5. The mutations found in the EG.5.1 reference sequence but not in the sequence of live viruses used in this study are indicated by asterisk following the residue numbers.

The mutations in the receptor binding domain (RBD) of the spike have dominant effect for the immune evasion ability since that is the main target for the neutralizing antibodies. The mutation in the RBD is increased and refined as the emergence of the variant over time (Figure 1A and 1B). The evolution of SARS-CoV-2 variants accompanied by the accumulated spike mutations are believed to be due to the selective pressure by neutralizing antibodies (10). Among the neutralizing antibodies elicited by SARS-CoV-2 infection and initial COVID-19 mRNA vaccines which use the ancestral SARS-CoV-2 spike sequence, only those targeting unchanged, common epitopes shared among the spikes of the omicrons and the earlier variants are effective for neutralization of the mutant variants as cross-neutralizing antibodies. It has been reported that boosted immunity established by repeated vaccination and/or infection contains cross neutralizing antibodies effective against Omicron variants BA.5, although they have significantly low activity against the XBB variants (11) probably due to the RBD mutations to modify effective neutralizing epitopes (Figure 1B). As EG.5.1 is reported to have further immune evasion ability (12), it is not known whether cross-reactive antibodies which can neutralize such variants with accumulated mutation are elicited at the beginning upon the primary infection by earlier variants or not.

In this study, the cross-neutralizing antibody titers of sera collected from patients experienced primary SARS-CoV-2 infection were assessed against live viruses of SARS-CoV-2 variants, D614G, Omicron BA.5, and EG.5.1. The expected difficulties to detect the low neutralizing titer due to the mutations in the Omicron variants were surmounted by using the severe or critical patients’ sera and by a time course evaluation method.

## MATERIALS AND METHODS

### Sera of COVID-19 patients and volunteers

Sera used in this study had been collected in our previous study (4) and stored in −80°C freezer. The background information of the sera is as follows. All patients were hospitalized and recruited at Hyogo Prefectural Kakogawa Medical Center in Hyogo, Japan. The patients were diagnosed with COVID-19 by PCR method which detects the SARS-CoV-2 genome in nasopharyngeal swab samples during April to May, 2020. The severity of COVID-19 patients were defined in the prior report: mild (symptomatic without evidence of pneumonia/hypoxia), moderate (pneumonia with oxygen saturation ≥90% on room air), severe (pneumonia with respirations above 30 per minute, severe respiratory distress, or oxygen saturation <90% on room air), and critical (severe symptom with additional need for mechanical ventilation) (4). Sera used in this study were selected from those of patients classified into severe and critical, which have been already confirmed to have high neutralizing activity against SARS-CoV-2 D614G (4).

Negative control sera had been collected from two healthy volunteers without SARS-CoV-2 infection or COVID-19 vaccination history at the time of blood collection. Positive control serum was collected from a volunteer who had been vaccinated twice with Sinovac (inactivated virus) followed by two times vaccination with Moderna (mRNA), and infected two times with mild symptom during June, 2021 and September, 2023. The ethical committee of Kobe University Graduate School of Medicine approved this study (approval code B200200). Written informed permissions were acquired from all participants and volunteers.

### Viruses and cells

The D614G mutant strain of SARS-CoV-2 was supplied from BIKEN Innovative Vaccine Research Alliance Laboratories in Osaka, Japan (DNA Data Bank of Japan [DDBJ]: accession no. LC644163). The SARS-CoV-2 variants BA.5 (EPI_13241867) was supplied by Japan’s National Institute of Infectious Diseases (Tokyo) and EG.5.1 (Hyo-23806755, EPI_ISL_19589168) was obtained from the Hyogo Prefectural Institute of Public Health Science. The EG.5.1 live virus used in this study was isolated on August 2023 when the EG.5.1 became prevalent in Japan, and contains mutations characteristic for EG.5.1, namely, Q52H, F456L, and F486P (12). As noted in Figure 1, the virus does not contain the mutations at L368I, S371F, S373P, S375F, T376A, D405N, R408N, and K417N which are reported in some other sequences of the EG.5.1 (e.g., EPI_ISL_17540065), keeping the same amino acid as the D614G variant. Note that EG.5.1 sequence lacking those mutations are also frequently found in the GISAID database (EPI_ISL_19621249, EPI_ISL_19621231, EPI_ISL_17558862, EPI_ISL_18134063 and so on). The viruses were propagated by infecting VeroE6 (TMPRSS2) cells (14) in Dulbecco’s modified Eagle medium (DMEM) supplemented with 2% fetal bovine serum (FBS) to establish a stock of each virus.

### Measurement of neutralizing activity against SARS-CoV-2

We performed a neutralization test in a biosafety level 3 (BSL3) facility in Kobe University Graduate School of Medicine to manipulate viruses of SARS-CoV-2 and assess the neutralizing activities of patients’ sera. Twenty-four hours prior to the experiment, 4 × 10^4^ VeroE6 (TMPRSS2) cells were inoculated into each well of 96-well tissue culture microplates. Samples of a twofold serial dilution of heat-inactivated (56°C, 30 min) sera were created utilizing DMEM. Virus stocks of SARS-CoV-2 D614G, Omicron BA.5, and EG.5.1 variants stored in −80°C freezer in the BSL3 facility were thawed just before the experiment, and the median tissue culture infectious dose (TCID50) of each virus stock were confirmed by an infection experiment in parallel to the neutralization assay. The diluted sera were combined with around 100 TCID50 of virus and incubated at 37°C for 1 hour. Subsequently, VeroE6 (TMPRSS2) cells in 96-well plates were inoculated with the serum-virus mixture and incubated at 37°C for 7 days. The cells were examined daily under a light microscope, and the neutralizing antibody titer was assessed as the highest serial dilution that did not display any cytopathic effects (CPEs). Because of the limitation of serum amount, we did not set multiple wells for each condition in one experiment. Experiments were done twice independently, and both showed similar results. The data of two experiments were not combined, and the data of one representative experiment is shown.

### Database and software

Sequence data of SARS-CoV-2 spike was obtained from the GISAID database (15). The illustration of spike protein is depicted using the UCSF ChimeraX 1.6 (16) using the BQ.1 spike coordinates (PDB ID: 8XI6) in the protein data bank (13). Kruskal-Wallis test and Dunn’s test were done by GraphPad Prism 8.

## RESULTS

### Sample information

This study focused on the sera of COVID-19 patients who were infected by SARS-CoV-2, and assessed the cross-neutralizing activities against Omicron variants. Information of patients were described in our previous study (4, 17) and summarized in Table I with information of controls for reference. Considering the infection period of the patients, April to May, 2020, it is assumed that all the patients infected by SARS-CoV-2 D614G variant which was prevalent in Japan at that time. Because relatively low neutralizing antibody titers against Omicron variants were expected, sera collected from COVID-19 severe/critically illness patients around 17–24 days post onset were selected, considering their high neutralizing antibody titers against D614G (4, 17). In total seven patients’ sera were used in this study: two from severe, and five from critical patients. The ages fall within the range from 57 to 78 years old at the infection timing, and 4 are males and 3 are females.

To evaluate the background neutralizing effect of human serum itself, sera from volunteers without SARS-CoV-2 infection or COVID-19 vaccination history were used. As a positive control, serum from a convalescent person who experienced twice of SARS-CoV-2 infection with mild symptom at the period when Delta and Omicron EG.5.1 variants were prevalent, and received two doses of mRNA vaccine with inactivated virus vaccine (Sinovac) and two doses of COVID-19 mRNA vaccine (Moderna), before and after the first infection, respectively. Because this person experienced multiple exposures to the SARS-CoV-2 antigens by infection and vaccination, the serum is expected to contain cross neutralizing antibodies against Omicron BA.5 and EG.5.1 considering our previous studies (11, 18).

### Sera from early COVID-19 patients have ability to neutralize Omicron variants BA.5 and EG.5.1

**Figure 2:**
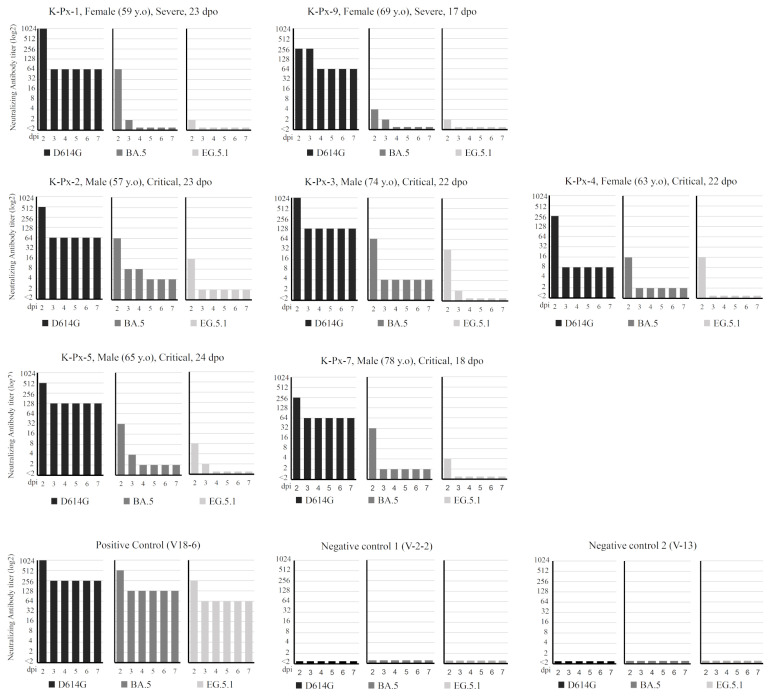
The neutralization assay results by sera with the information of patients’ age, disease severity, and blood collection timing as days post onset (dpo). As indicated, K-Px-1, and 9 are the severe COVID-19 patients and K-Px-2, 3, 4, 5, and 7 are the critical patients. Results for the positive control serum (V18-6) and the negative control sera (V-2-2 and V-13) were also included. Vertical axes show the neutralizing antibody titer in log2 scale, and the horizontal axes show the day after infection (dpi). Each contains three plots for D614G, Omicron BA.5, and EG.5.1.

The main question of this study is whether the sera from individuals infected with the early SARS-CoV-2 variant D614G have neutralizing antibodies which are effective against Omicron variants, BA.5 and EG.5.1. CPEs by these viruses were observed in the wells without serum by 2 dpi (data not shown). The sera from COVID-19 patients exhibited neutralization at 2 dpi in a dilution dependent manner. The maximum dilutions in which CPEs were not found were shown as the neutralizing antibody titers, and plotted in Figure 2. The titers decreased over time from 2 to 3 dpi, further declined at 4 dpi, then become converged, indicating that the virus infection and inhibition by the sera occurred mainly for 4 days and became equilibrated or stable state in the experimental condition. The control sera obtained from the volunteers without COVID-19 history could not inhibit the infections even at the highest concentration, showing CPEs at 2 dpi (Figure 2).

Against D614G, all sera showed relatively high neutralization titers even after 4 dpi. The neutralizing titers range from 256 to 1024 at 2 dpi and from 8 to 128 at 4 dpi (Figure 2). The neutralization titers for BA.5 were relatively low at all the time points in comparison with those for D614G, especially after 3 dpi. When the sera were categorized based on the patients’ severity, there were differences in the neutralizing activity between sera from severe and critical patients. Sera from severe patients exhibited neutralizing activity against BA.5 at 2 dpi, but intriguingly, after 4 dpi they lost the capacity to neutralize BA.5. On the other hand, all sera from critical patients exhibited inhibition for BA.5 for 7 dpi. For EG.5.1, neutralizing activities were shown at 2 dpi (Figure 2), albeit relatively low compared to those for other examined viruses; nevertheless, all sera lost this capability in the subsequent days, with one exception of the sera from the patient K-Px-2. For EG.5.1, it was observed the disparity in greater titers for critical patients compared to severe patients at 2 dpi.

The positive control sera from the volunteer V18-6 who experienced vaccination and infection exhibited robust neutralizing activity for all viruses at all the time points, showing the difference between humoral immunities induced by primary infection and that induced after repeated stimulations by SARS-CoV-2 antigens as boost.

### Trend of correlation between neutralizing activities against the three variants

**Figure 3:**
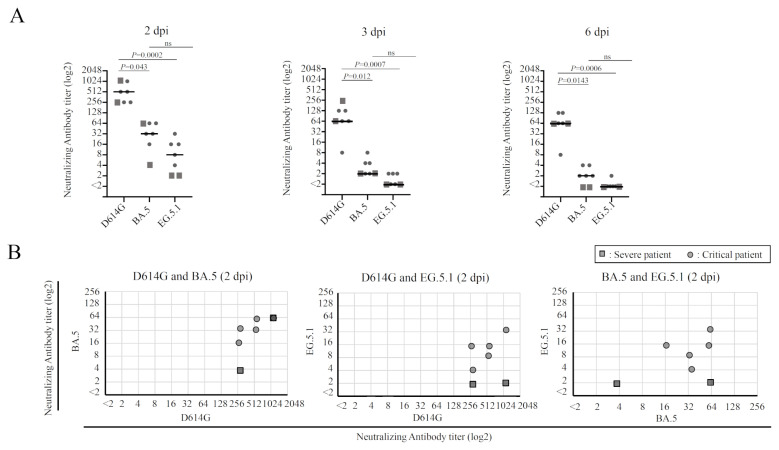
Sub-analysis of the neutralization assay results against D614G, BA.5, and EG.5.1. **A**. Comparison of neutralizing antibody titers against D614G, Omicron BA.5, and EG.5.1. Statistics analysis was performed by Kruskal-Wallis test followed by Dunn’s test. The *P* values are shown if they were <0.05 otherwise indicated as not significant (ns). The plots were prepared for the three time points, 2 dpi, 3 dpi and 6 dpi. The vertical axis stands for the neutralizing antibody titer (log2). The median lines are added for each category. **B**. Correlation between the neutralizing antibody titers at 2 dpi against D614G and BA.5, D614G and EG.5.1, and BA.5 and EG.5.1. The vertical and horizontal axes are the neutralizing antibody titer (log2). The circle and square dots represent the data of critical and severe patient’s sera, respectively.

Next, the data of neutralizing assay shown in Figure 2 were combined to analyze the trend. Figure 3A illustrates the neutralization titers for the three variants at three distinct time points, that is, 2, 3, and 6 dpi. Indeed, 6 dpi is a standard time point for assessing neutralizing antibody titers in our previous studies (5, 11, 17, 19, 20). There is a trend that the sera have the highest neutralizing titer against D614G, and relatively low neutralizing titers against Omicron BA.5 and EG.5.1 at all the time points (Figure 3A). The differences between neutralizing titers for BA.5 and EG.5.1 are not significant. The fold difference of median titers between D614G and BA.5 or EG.5.1 were 16 or 64 folds at 2 dpi, respectively. The fold difference between neutralizing titers for D614G and BA.5 was slightly increased at 3 dpi and 6 dpi as the difference became 32 folds at both time points. The change could not be traced for the EG.5.1 since CPEs appeared in the wells after 3 dpi.

The correlations between the neutralization titers against D614G and BA.5, D614G and EG.5.1, and BA.5 and EG.5.1 were analyzed in Figure 3B. A positive correlation between the neutralizing titers is observed. Higher neutralizing titers against D614G are basically accompanied by higher titers against BA.5 or EG.5.1. Similar correlation is observed for neutralizing titers of BA.5 and EG.5.1. It may be noteworthy that the points for sera from critical patients form clusters (Figure 3B, grey dots), while the points for severe patients’ sera tend to be located outside the clusters (Figure 3B, grey square).

## DISCUSSION

The sera elicited by SARS-CoV-2 primary infection were demonstrated to contain cross neutralizing antibodies effective even for Omicron EG.5.1 variant. Neutralizing antibody titers of sera from COVID-19 patients naturally infected by early SARS-CoV-2 variants D614G generally were not analyzed in detail against Omicron variants, since such lately-emerged SARS-CoV-2 variants have high immune evasion ability, hence it is expected that neutralizing antibodies are difficult to be detected for the primary immunity without boost. The difficulty was overcome here by focusing on the severe/critical patients’ sera at two to three weeks post onset when the neutralizing titers reach a peak (4), and by detecting early protection effect at 2 dpi. In previous studies, the neutralization titers were defined by refereeing to the emergence of CPEs at 6 dpi (5, 11, 17, 19, 20). Evaluation of neutralizing antibody titer at the time point of 2 dpi or earlier is widely used in focus reduction neutralizing test (FRNT) (21, 22) in which live virus infection and its neutralization by sera are determined by detecting SARS-CoV-2 antigens in infected cells. The weak protecting activities were missed owing to the evaluation at a late and single time point.

The neutralizing activities of sera against EG.5.1 did not last for 3 dpi or longer (Figure 2). Nonetheless they showed clear protecting activity at 2 dpi in low dilution conditions, and apparently slowed down the progression of CPE formation by the virus. As far as we know, there is no widely accepted correlation linking neutralizing antibody titers determined by in vitro assays and their clinical significance in actual persons. Nonetheless, it is tempting to speculate the meaning of the observed neutralization effect as follows. Considering the actual immune response in individuals who have such humoral immunity, the partial neutralization effect by serum antibody seems to have important contribution to counteract viruses; the delay in infection procedure is critical because it gives chance of clearance by immune systems, and memory B cells producing the broadly neutralizing antibodies as well as memory T-cells will awake in responding to the antigens, leading to boosted immunity based on the acquired immunity at the primary infection. Although reinfection or breakthrough infection of SARS-CoV-2 variants are frequently reported (23, 24), it may contribute to alleviate the symptoms.

The observed difference in neutralizing activities against Omicron BA.5 and EG.5.1 (Figures 2 and 3A) indicated that a substantial portion of the cross-neutralizing antibodies targets the neutralizing epitopes on the RBD. The EG.5.1 live virus used in this study does not contain eight mutations in the RBD when compared with some EG.5.1 sequences in the GISAID database (15) (Figure 1A, asterisk), nonetheless it showed higher immune evasion ability probably because those amino acid residues are not involved in the target sites for major neutralizing antibodies around the receptor binding site (25) (top side of RBD in Figure 1B). Although most of the lacking mutations have little effect in immune evasion, K417N is reported to have some contribution to the immune evasion (26). Thus, it is possible that EG.5.1 with those eight mutations may show further immune evasion. The RBD of BA.5 remains the known binding site for neutralizing antibodies around the amino acid residues R346 and V445 (27), while the XBB and the descendant have mutation around this area (28) (Figure 1B). Our prior research exploring broadly neutralizing monoclonal antibodies from B cells of individuals who received a two-dose mRNA vaccine following infection with D614G led to identification of a broad neutralizing monoclonal antibody MO1 which targets around the R346 of RBD (29). The MO1 could neutralize BA.5 but inactive against variants appeared after that, such as XBB. The decrement of neutralizing activities against EG.5.1 compared with those against BA.5 is explained by existence of MO1-like antibodies in the sera, which contribute to the neutralization of SARS-CoV-2 variants up to BA.5, but loose activity against EG.5.1 due to the additional mutation on the RBD. Interestingly, the critical COVID-19 patients’ sera contain BA.5 neutralizing antibodies sufficient for preventing CPE formation for 7 dpi, while the severe COVID-19 patients’ sera do not (Figure 1). Relationship with the elevated cytokine/chemokine level observed for the critical patients (4) may be implied, although more detailed study with statistical analysis is needed for further discussion. The virus multiplication is considered to be large. In these cases, it is also thought that neutralizing antibody-producing B cells that recognize a wider range of epitopes have been formed.

Omicron XBB and descendants including EG.5.1 can escape from most of neutralizing antibodies targeting RBD, except for some rare antibodies (29, 30). The detected neutralizing activities against the EG.5.1 could be explained by the antibodies targeting subdomain 1 (SD1) or S2 domain where no or quite few numbers of mutations are found even in the EG.5.1 (Figure 1A), although the neutralizing epitopes on those domains are limited. One example of anti-SD1 antibodies is the neutralizing antibody MO11 which was identified in our previous study from the B cells of the convalescent patients who received subsequent three doses of mRNA vaccination (13). As the donor of the cells is one of the COVID-19 follow-up patients, it is tempting to speculate that the immune response upon the primary infection leads to production of various B cells including those secreting MO11-like SD1 targeting antibodies as a part of initial humoral immunity against SARS-CoV-2.

This study is limited by the small sample size, n = 2 for severe patients, and n = 5 for critical patients, hindering the potential for generalization. Although neutralizing titers against the EG.5.1 could be evaluated, the detection sensitivity is still low to perform numerical comparison analysis in detail. As the sera were selected only from severe and critical COVID-19 patients, the relationship between the COVID-19 severity and the induction of cross neutralization antibodies at the primary infection could not be analyzed without involvement of mild and moderate patients’ sera. Improvement in the sample number and the experimental condition will be needed in future study to clarify the observation.

We reported that when severe or critical patients infected with D614G were vaccinated, neutralizing antibodies against the Omicron variants were also induced (18). This study indirectly proves that the initial infection with D614G has formed antibody-producing cells that can recognize common epitopes, albeit in very small amounts, and that they have been boosted by vaccination.

## Figures and Tables

**Table I:** Information about patients who provided the sera

Sample group	Code	Sex, Age*	Severity†	Infection period	Serum collection (dpo‡)	Vaccination
Test samples	K-Px-1	F, 59	Severe	April, 2020	23	-
	K-Px-9	F, 69	Severe	May, 2020	17	-
	K-Px-2	M, 57	Critical	April, 2020	23	-
	K-Px-3	M, 74	Critical	April, 2020	22	-
	K-Px-4	F, 63	Critical	April, 2020	22	-
	K-Px-5	M, 65	Critical	April, 2020	24	-
	K-Px-7	M, 78	Critical	May, 2020	18	-
Negative controls	V-13	F, 39	-	-	2021/7/6	-
	V-2-2	M, 42	-	-	2021/5/25	-
Positive control	V-18-6	F, 31	Mild/Mild	June, 2021/September, 2023	2023/12/19	2 doses Sinovac, 2 doses Moderna

* Sex: F, female; M, male; Age is at the sample collection date.

† Severity was defined in previous study as follows: Severe: require of supplemental oxygen; Critical: requirement of intensive care, including mechanical ventilation. Upper respiratory symptoms include fever, cough, and sore throat.

‡ dpo: days post onset.
